# Reducing delay to endovascular reperfusion after relocating a thrombolysis unit

**DOI:** 10.3389/fneur.2022.989607

**Published:** 2022-09-23

**Authors:** Nicolaj Grønbæk Laugesen, Klaus Hansen, Joan Højgaard, Helle Klingenberg Iversen, Thomas Truelsen

**Affiliations:** ^1^Department of Neurology, Stroke Center Rigshospitalet, Rigshospitalet, Copenhagen, Denmark; ^2^Faculty of Health Sciences, University of Copenhagen, Copenhagen, Denmark

**Keywords:** stroke, thrombectomy, reperfusion, in-hospital delay, revascularisation

## Abstract

**Background and aims:**

Functional outcome following mechanical thrombectomy (MT) in patients with acute ischemic stroke and large vessel occlusion is time-dependent and worsens with increasing delay. Time to endovascular reperfusion is potentially modifiable with changes in organizational structure. We investigated the changes in time to reperfusion of relocating the intravenous thrombolysis (IVT) services from a non-MT center to a MT-capable center.

**Methods:**

We present an observational, consecutive, retrospective, single-center cohort study of 253 stroke patients treated with MT, 2017–2019. The observation period was divided into before and after the relocation of IVT services in 2018, period 1 and period 2, respectively. The two hospitals were located 13 km apart in an urban area, and following the relocation, IVT was administered at the MT-capable center. Time metrics were registered and divided into two main intervals, namely, ambulance departure from stroke onset location to imaging (ambulance-imaging) and imaging to reperfusion (imaging-reperfusion). The interval imaging-reperfusion included inter-hospital transfer to the MT-capable center in period 1. The association of the imaging-reperfusion duration and functional outcome at 90 days was analyzed using ordinal logistic regression.

**Results:**

No significant change in ambulance-imaging was observed from a median of 27 min (interquartile range [IQR] 22–37) in period 1 to 30 min (IQR 23–40) in period 2, *p* = 0.19, while the median time of imaging-reperfusion decreased from 173 min (IQR 137–230) to 114 min (IQR 84–152), *p* < 0.001. The largest absolute time reduction from imaging to reperfusion was seen from imaging to arrival at the angio suite from 89 min (IQR 76–111) to 42 min (IQR 28–63), *p* < 0.001, which included inter-hospital transfer in period 1. In multivariate analysis, every 10 min of increased delay from imaging to reperfusion was associated with poorer functional outcome with an adjusted odds ratio of 0.95 (95% CI: 0.95–0.98), *p* < 0.001.

**Conclusion:**

Relocation of IVT services to an MT-capable center was the main cause of reduced time to reperfusion for patients treated with MT and was implemented without affecting prehospital transportation time. These results suggest that patient outcome can be improved by optimizing the organization of IVT and MT services in urban areas.

## Introduction

Randomized clinical trials have shown the benefit of acute mechanical thrombectomy (MT) in the treatment of acute ischemic stroke with large vessel occlusion (LVO) and are now considered standard care (1–6). Factors associated with the outcome following MT include non-modifiable factors, such as age or stroke symptoms severity, whereas time from onset to reperfusion is potentially modifiable (7–10). The impact on the functional outcome of time from onset to reperfusion has been studied; however, once patients are scanned and found eligible for acute MT, less is known about the impact delay from imaging to reperfusion (11–13). From studies on treatment with intravenous thrombolysis (IVT), workflow optimizations have been shown to reduce delay at every interval and improve the outcome of patients (14).

One proposed strategy for reducing time to reperfusion in MT is to bypass IVT stroke units without MT-capability (non-MT centers) for patients with suspected LVO and directly admit these selected patients to an MT-capable center (15). However, the recently published RACECAT study did not show the benefit of direct admission to an MT-capable center (16). Based on modeling data, another study suggested that in urban areas, patients with suspected LVO should be admitted directly to an MT-capable center if the transportation time did not increase more than 30 min compared to the transportation time to the nearest non-MT center (17).

In this study, we investigated the effect of relocating IVT services from a non-MT center to an MT-capable center in an urban setting with the same catchment area, and whether this organizational change was associated with reduced time to MT and with functional outcome at 90 days.

## Materials and methods

### Study population

The IVT services were relocated from a non-MT center to an MT-capable center on 17 April 2018; the two facilities were positioned 13 km apart in an urban area. Before the relocation of the IVT services, the non-MT center served as a primary stroke center for the catchment area (1.8 million inhabitants, census 2018), where all patients with suspected acute ischemic stroke were admitted for initial examination and potential treatment with IVT, and patients were only transferred to the MT-center if an LVO was identified. For the identification of LVO, either computed tomography (CT)-angiography or magnetic resonance (MR)-angiography was used. Prior to the relocation, no patients were acutely admitted for IVT treatment at the MT-capable center, and following the relocation, IVT was no longer provided at the non-MT center. The catchment area remained the same before and after the relocation, and only the location of IVT services was altered. Emergency Medical Services (EMS) in Denmark is an integral part of the universal healthcare system as a single provider of prehospital services for all citizens. Following dispatch, if EMS personnel suspected the patient of acute stroke, the neurologist on call at the IVT services was contacted for consultation and possible acute admission. All patients would be admitted directly to the scanning room for acute stroke imaging during the entire observation period and at both hospitals.

At both hospitals, IVT was administered in the scanner room immediately following imaging and within 4½ h after symptoms onset according to international guidelines (18). Imaging sequences acquired at the non-MT center in period 1 were transferred electronically for evaluation at the MT center. Assessment of eligibility for MT was shared by a vascular neurologist and a neurointerventionist. There was no upper age limit, but relative contraindications included pre-stroke modified Rankin Scale (mRS) score of ≥3, comorbidities that could increase the risk of procedural complications and infarct volume >1/3 of the middle cerebral artery territory, and clinical/imaging mismatch should be suggestive of salvageable tissue. The procedure was performed at the discretion of the neurointerventionist and included the use of thrombus aspiration and stent retrievers (devices used included Solitaire stent, Medtronic, Minneapolis, MN, USA; EmboTrap, Neuravi, Galway, Ireland; ERIC, MicroVention Terumo, Aliso Viejo, CA, USA; pReset, Phenox, Bochum, Germany; Capture, Medtronic, Minneapolis, MN, USA), and intraarterial thrombolytics. Patients referred for EVT from other IVT stroke units were not included in the analyses.

The observation period was divided into two periods: period 1 from 1 January 2017 to 16 April 2018, and period 2 from 17 April 2018 to 31 December 2019 (before and after the relocation). Adult patients were consecutively included, and the following data were obtained: sex (male or female), age (years), time of stroke symptoms onset registered as either known/witnessed or as when the patient last seen well (LSW), pre-stroke mRS, pre-procedural National Institute of Health Stroke Scale (NIHSS), and, at 24 h following EVT, reperfusion results in the modified treatment in cerebral infarction (mTICI) score ranging from 0 (no reperfusion) to 3 (full reperfusion) as assigned by the attending neurointerventionist, and treatment with IVT (yes/no).

**Figure 1:**
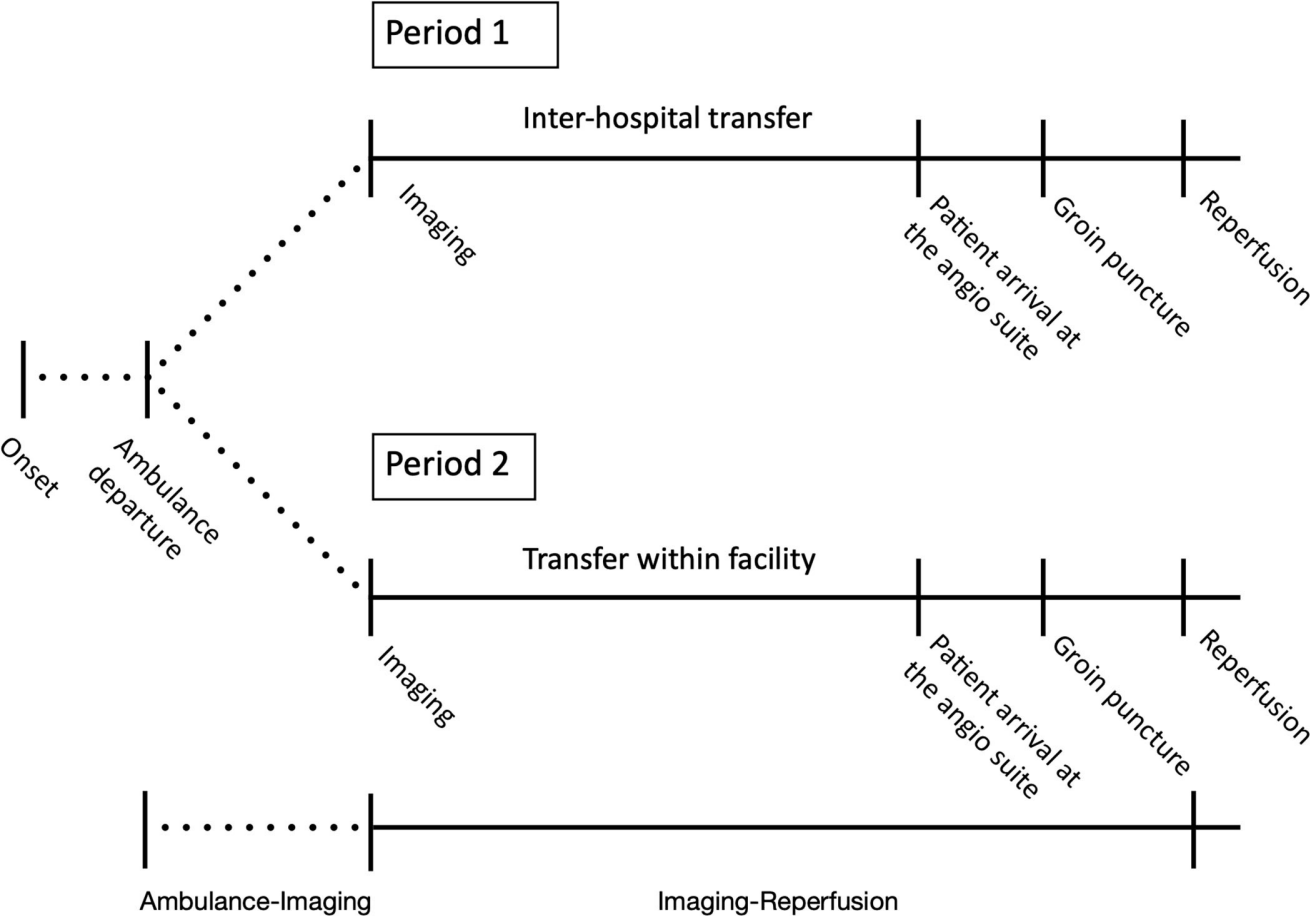
Changed patient flow following the relocation of IVT services. Period 1 corresponded to before the relocation of the IVT services (1 January 2017–16 April 2018) and period 2 to after the relocation (17 April 2018–31 December 2018). In period 1, IVT services were located at a non-MT center, and in period 2, IVT services were located at an MT-capable center. Time metrics were divided into time from ambulance departure from stroke onset location to first imaging after admission at the first hospital (ambulance-imaging) and from first imaging after admission to reperfusion (imaging-reperfusion). In period 1, imaging-reperfusion included inter-hospital transfer.

Time metrics were divided into time from ambulance departure from stroke onset location to first imaging (ambulance-imaging) and from imaging to reperfusion (imaging-reperfusion) (Figure 1). The time of ambulance departure was retrospectively collected from emergency medical service records. Time of imaging was defined as the automatic time label applied by imaging software on the first imaging sequence of either CT-angiography or MR-angiography. Reperfusion was defined as the time of satisfactory angiographic result achieved or as the time of procedure termination when no reperfusion was achieved (mTICI 0), or if no LVO was visualized on the first run of the digital subtraction angiography. Evaluation of mRS at 90 days follow-up was performed at the outpatient clinic of the MT-capable center by trained neurovascular clinicians.

This study was approved by appropriate authorities, Danish Patient Safety Authority, and the Center for Regional Development (Reference R-21010059) and was exempted from approval by the ethics committee.

### Outcomes

The primary analysis was for changes in time of ambulance-imaging and imaging-reperfusion between period 1 and period 2. Secondary analysis was functional outcome using mRS with favorable outcome defined as mRS 0–2 and non-favorable outcome defined as mRS 3–6. Furthermore, we analyzed the association of time in imaging-reperfusion with multivariate shift analysis of mRS at 90 days.

### Statistics

For patient characteristics, continuous variables were summarized as medians with interquartile ranges (IQR), and categorical variables were presented as frequencies with percentages. For continuous variables, differences were assessed using Wilcoxon-Mann-Whitney U test. For categorical variables, the Pearson χ^2^ test was used to compare the distributions between groups.

The association of imaging-reperfusion in 10-min increments with shifts in functional outcome (mRS) at 90 days was analyzed by means of ordinal logistic regression adjusted for age (continuous), sex (male/female), NIHSS, general anesthesia or consciousness sedation, registration as known onset or last seen well, pretreatment with IVT (yes/no), and observational period (period 1/period 2). The level of statistical significance was set at *p* < 0.05. The statistical analyses were performed using IBM SPSS Statistics 28.

## Results

During the observation period, a total of 712 patients had groin puncture for MT, and of these, 253 patients were included for analysis as the remaining 459 patients were referred from other primary stroke centers not participating in this study. A total of 73 patients were admitted to the non-MT center in period 1, and 180 patients were directly admitted to the MT-capable center in period 2 (Table 1). Baseline characteristics were evenly distributed but differed significantly with regard to the proportion of patients receiving pre-treatment with IVT, which decreased from 49 (67.1%) in period 1 to 83 patients (46.1%) in period 2, *p* = 0.002 (Table 1). From period 1 to period 2, there was a significant increase in time from stroke symptoms onset to ambulance departure from a median of 60 min (IQR 30–159) to 97 min (IQR 38–352), *p* = 0.02, predominantly driven by LSW patients (Table 2).

**Table 1:** Baseline characteristics.

	**Period 1** **(*n =* 73)**	**Period 2** **(*n =* 180)**	***p*** **-value**
Age, years, median (IQR)	72 (62–80)	73 (62–79)	0.82
Sex (female), *n* (%)	33 (45.2%)	77 (42.8%)	0.72
Known time of onset, *n* (%)	48 (65.8%)	107 (59.4%)	0.35
Pretreatment with IVT, *n* (%)	49 (67.1%)	82 (45.6%)	<0.01
Procedure in general anesthesia, *n* (%)	56 (76.7%)	153 (85.0%)	0.12
NIHSS at baseline, median (IQR)	16 (10–19)	17 (10–21)	0.73
NIHSS at 24 hours, median (IQR)	8 (3–17)	8 (3–16)	0.93
Pre-stroke mRS 0–2, *n* (%)	71 (97.3%)	169 (93.9%)	0.27
**Occlusion site**, ***n*** **(%)**			
Anterior circulation	64 (87.7%)	153 (85.0%)	0.58
Tandem occlusion	10 (13.7%)	31 (17.2%)	
ICA	13 (17.8%)	27 (15.0%)	
M1	33 (45.2%)	64 (35.6%)	
M2	8 (11.0%)	30 (16.7%)	
Posterior circulation	6 (8.2%)	20 (11.1%)	0.49
No occlusion	3 (4.1%)	7 (3.9%)	0.94
**Reperfusion result of MT in mTICI**, ***n*** **(%)**^***†***^			
Successful reperfusion (2b-3)	53 (72.6%)	141 (78.3%)	0.31

† Patients with no occlusion were excluded.

Tandem occlusion, concurrent occlusions of the ICA and of the MCA; No occlusion, satisfactory reperfusion result on the first angiographic run following groin puncture.

IVT, intravenous thrombolysis, NIHSS, National Institute of Health Stroke Scale, mRS, Modified Rankin Scale, ICA, internal carotid artery, M1, first segment of the middle cerebral artery (MCA), M2, proximal second segment of the MCA, MT, mechanical thrombectomy, mTICI, modified treatment in cerebral infarction.

**Table 2:** Logistical measures.

	**Period 1**	**Period 2**	***p*** **-value**
Onset to reperfusion	290 (225–406)	298 (177–674)	0.87
Onset to ambulance^*†*^	60 (30–159)	97 (38–352)	0.02
Known onset	39 (28–65)	48 (26–97)	0.21
Last seen well	192 (121–477)	482 (208–750)	<0.01
Ambulance-imaging^*†*^	27 (22–37)	30 (23–40)	0.19
Ambulance to door^*†*^	20 (12–24)	15 (10–23)	0.13
Door to imaging	9 (6–12)	12 (8–18)	<0.001
Imaging-reperfusion	173 (137–230)	114 (84–152)	<0.001
Imaging to arrival at angio suite	89 (76–111)	42 (28–63)	<0.001
Arrival at angio suite to groin puncture	22 (17–32)	26 (18–35)	0.13
Groin puncture to reperfusion	52 (24–77)	34 (20–55)	<0.01

Values are in minutes; median (IQR).

† Ambulance departure missing for 7 patients (all in period 2).

Definition of time points: Onset, onset of stroke symptoms; Reperfusion, satisfactory angiographic result achieved or termination of the procedure if mTICI 0 or no occlusion on at first angiographic run; Ambulance, ambulance departure from stroke onset location; Door, patient arrival at the non-MT center in period 1 and at the MT-capable center in period 2; Needle, administration of IVT; Imaging, time of automated time label applied by imaging software of first imaging sequence after admission; Arrival at angio suite, patient arrival at the angio suite of the MT-capable center; Groin puncture, groin puncture for intended MT.

mTICI, modified treatment in cerebral infarction, LVO, large vessel occlusion, IVT, intravenous thrombolysis, MT, mechanical thrombectomy.

### Time from ambulance departure to imaging

The duration of the interval ambulance-imaging remained stable, median 27 min (IQR 22–37) in period 1 and 30 min (IQR 23–40) in period 2, *p* = 0.19 (Table 2). Median time from ambulance departure to door decreased from 20 (IQR 12–24) to 15 (IQR 10–23), although not statistically significant, *p* = 0.13. We observed an increase in door to imaging, median 9 min (IQR 6–12) to 12 min (IQR 8–18), *p* < 0.01.

### Imaging to reperfusion

The duration of imaging-reperfusion decreased from period 1 to period 2, median 173 min (IQR 137–230) to 114 min (IQR 84–152), *p* < 0.001 (Table 2). The largest absolute reduction was from imaging to patient arrival at the angio suite from a median of 89 min (IQR 76–111) in period 1 to 42 min (IQR 28–63), *p* < 0.001. In period 1, this interval included the inter-hospital transfer from the non-MT center to the MT-capable center, while no inter-hospital transfer was necessary in period 2 as IVT and MT were provided within the same center. A significant reduction was also observed in procedural time (from groin puncture to reperfusion) from a median of 52 min (IQR 24–77) in period 1 to 34 min (IQR 20–55) in period 2, *p* < 0.01.

There was no observed difference in time from patient arrival at the angio suite to groin puncture between patients receiving general anesthesia compared to conscious sedation, median 25 min (IQR 18–34) and 24 min (IQR 16–35), *p* = 0.46, respectively.

In the subpopulation of patients pretreated with IVT [49 patients (67.1%) in period 1 and 82 patients (45.6%) in period 2], the time from door to needle increased from a median of 17 min (IQR 13–26) to 22 min (IQR 16–32), *p* = 0.022; median door to needle time was 17 min (IQR 12–26) in 2017, 16 min (IQR 13–23) in 2018, and 25 min (IQR 19–36) in 2019.

### Functional outcome at 90 days

There was no difference in the proportion of patients achieving favorable outcomes (mRS 0–2) between the two periods (54.8% in period 1 and 45.0% in period 2, *p* = 0.32) (Table 3). Mortality remained stable at 19.2% in period 1 and 23.3% in period 2, *p* = 0.34.

**Table 3:** Functional outcome at 90 days.

	**Period 1** **(*n =* 73)**	**Period 2** **(*n =* 180)**	***p*** **-value**
Favorable outcome (mRS 0–2)	40 (54.8%)	81 (45.0%)	0.32
Mortality (mRS 6)	14 (19.2%)	42 (23.3%)	0.34
Missing (lost to follow-up)	1 (1.4%)	13 (7.2%)	
Alive at 90 days with missing functional status^*†*^	1 (1.4%)	9 (5.0%)	

Values are frequencies (%).

† Patients lost to follow-up with vital status confirmed through linkage to the National Danish Civil Registration Services using a unique identification number assigned to all Danish citizens.

mRS, modified Rankin Scale.

In multivariate analysis, every 10-min increase in time from imaging to reperfusion was associated with a shift toward poor functional outcome, aOR = 0.95 (95% CI: 0.93–0.98), *p* < 0.001 (Table 4). Only two other variables were also significantly associated with functional outcomes in multivariate analysis; NIHSS at baseline, aOR = 0.90 (95% CI: 0.87–0.93), *p* < 0.001, and age, aOR = 0.96 (95% CI: 0.94–0.98), *p* < 0.001.

**Table 4:** Delay from imaging to reperfusion and functional outcome.

	**Univariate analysis**			**Multivariate analysis**		
	**OR**	**95% CI**	***p*** **-value**	**aOR**	**95% CI**	***p*** **-value**
Age	0.961	0.945–0.977	<0.001	0.961	0.944–0.978	<0.001
Sex	0.819	0.522–1.285	0.38	0.845	0.522–1.366	0.49
Observation period	0.696	0.427–1.133	0.15	0.652	0.378–1.125	0.12
General anesthesia	0.395	0.220–0.711	<0.01	0.705	0.376–1.321	0.28
Known onset	2.149	1.348–3.426	<0.01	1.451	0.866–2.431	0.16
Pretreatment with IVT	2.417	1.530–3.820	<0.001	1.648	0.993–2.734	0.05
NIHSS at baseline	0.908	0.879–0.937	<0.001	0.903	0.872–0.934	<0.001
Imaging-reperfusion (10-minute increments)	0.979	0.954–1.004	0.10	0.952	0.925–0.979	<0.001

The association of patient characteristics and logistical measures with ordinal shifts in mRS at 90 days.

Variables: Age (years, continuous), sex (female), observation period (period 2), general anesthesia compared to conscious sedation, known onset compared to last seen well, pretreatment with IVT (yes/no), NIHSS at baseline (continuous), time from imaging to reperfusion in 10-min increments.

OR, Odds ratio; aOR, adjusted odds ratio; CI, confidence intervals; IVT, intravenous thrombolysis; NIHSS, National Institute of Health Stroke Scale.

A subanalysis in patients with a known time of onset [48 patients (65.8%) in period 1 and 107 patients (59.4%) in period 2], every 10-min delay from onset to reperfusion, increased the risk of poor outcome, aOR = 0.97 (95% CI: 0.95–0.99), *p* = 0.03; also in this population, time from imaging to reperfusion was significantly associated with outcome, aOR = 0.95 (95% CI: 0.92–0.98), *p* < 0.01.

## Discussion

This study shows that relocation of the IVT services from a non-MT center to an MT-capable center, located 13 km apart in an urban environment, with no change in the catchment area, was significantly associated with reduced delay to reperfusion without influencing prehospital ambulance transportation time. A reduction in the median time from imaging to patient arrival at the angio suite from 89 to 42 min was the main driver of the reduction from imaging to reperfusion. Multivariate analyses showed that increasing delay from imaging to reperfusion was significantly associated with the risk of poor functional outcome at 90 days.

Previous studies have shown that outcome following MT is time-dependent but predominantly focused on the total duration from stroke symptoms onset to reperfusion (11–13, 19). However, once a patient is scanned and is eligible for MT with potentially salvageable tissue, every effort should be done to reduce delay to MT, irrespective of when stroke symptoms started—“the clock is re-set.”

The organizational change of relocating IVT services from a non-MT center to an MT-capable center, thereby avoiding inter-hospital transfer, could potentially increase prehospital transportation time and negate any time saved. Few studies have reported inter-hospital transfer in MT but in the American STRATIS registry the authors reported longer time from stroke symptoms onset to reperfusion for patients first admitted to a non-MT center (median 311 min) than for patients directly admitted to an MT-capable center (median 202 min); prehospital transportation time was unchanged, like in our study, which is likely generalizable to other urban areas with short inter-hospital distances (19).

The non-MT center, where IVT treatment was provided in period 1, and the MT-capable center were organized under the same hospital administration with staff working at both facilities. There was an increase in median time from door to needle (IVT treatment) between the periods, mainly driven by data from 2019. This corresponds to data from the Danish Stroke Registry on all patients treated with IVT in the capital region of Denmark, where the median door-to-needle time increased from 19 min in 2018 to 24 min in 2019 (20). This is likely driven by the implementation of results from the WAKE-UP trial, which enabled IVT treatment in patients with unknown time of onset based on MRI, which is more time-consuming compared to CT (21). The IVT services were relocated to the MT-capable center in 2018, before the results of the WAKE-UP trial, and in that year, the door-to-needle-time was unchanged suggesting that the relocation of IVT services did not negatively impact door-to-needle time.

### Changes in patients' admittance and treatment with mechanical thrombectomy

The comparison of the two periods may be biased due to changes in patient characteristics and the provision of healthcare services. During our observation period, trial results extended the time window for MT, and patients with unknown time of stroke symptoms onset were examined for potential IVT treatment based on MRI, which increased the number of patients being acutely admitted (21–23). This change in admission policy is consistent with data from our study as the proportion of patients treated with IVT decreased significantly, and there was a significant increase in time from stroke symptoms onset to ambulance departure. In our analysis, we adjusted for pretreatment with IVT, which had no effect on the outcome. From period 1 to period 2, the number of patients treated with MT increased, while the catchment area was unchanged suggesting improved awareness of the eligibility criteria for MT and extension of time for MT rather than changes in LVO occurrence. This could possibly influence the outcome of patients in period 2 negatively with more patients arriving in the late time window. We, therefore, included adjustment for the study period to reduce the risk of residual bias according to when the MT procedure was performed, but this variable was not significantly associated with the mRS at 90 days.

In time metrics analysis, time from groin puncture to reperfusion also decreased significantly from 52 to 34 min, without a change in NIHSS severity at baseline or location of vessel occlusion, and with the same reperfusion results. Increased experience by neurointerventionists is a likely contributing factor in reducing the overall delay to reperfusion (24). However, in our study, this interval constituted less than one-third of the overall time reduction from imaging to reperfusion.

## Strengths and limitations

Strengths of our study include the consecutive, observational design with complete registration and follow-up regarding the vital status of all MT-treated patients for the catchment area, which is mandatory by the Danish Health Authorities, and MT is only performed as a public health service. We have detailed information on time from ambulance departure to reperfusion.

However, our study also has limitations, as patient composition changed over time following the publication of results from WAKE-UP and trials on MT in extended time windows (21–23). We find it unlikely that these changes could influence the time registrations that were based on automatic and standardized data registrations only. Although the observation period was not significantly associated with functional outcomes in our analysis, it is still possible that other factors not accounted for could contribute to the observed changes in outcome. However, other studies have shown that increasing time to reperfusion is associated with a worse outcome, and results were consistent in the subanalysis of patients with a known time of stroke symptoms onset. The study is not a randomized clinical trial, but an analysis of real-life data using the same catchment area before and after an organizational change, which did not impact prehospital transportation time, but in other settings, such changes may cause unwanted delay. The reperfusion results were assessed by the attending neurointerventionist, and the evaluation of functional outcome was performed by clinicians who had full access to the patient's files, but none of this would impact the time registrations. Finally, our data do not allow for the estimation of numbers needed to treat as a result of the reduction in delay of imaging-reperfusion.

In conclusion, in our population, relocating IVT services to an MT-capable center was the main cause of reduced delay to reperfusion in patients with acute ischemic stroke and LVO, without influencing prehospital transportation time. Time from imaging to reperfusion was a significant predictor of functional outcome at 90 days following MT, and our study suggests that this organizational change may likely benefit patients eligible for acute MT in other urban areas if prehospital time is not prolonged.

## Data availability statement

The datasets presented in this article are not readily available because individual patient data cannot be made accessible according to Danish law. Requests to access the datasets should be directed to nicolaj.groenbaek.laugesen.01@regionh.dk.

## Ethics statement

Ethical review and approval was not required for the study on human participants in accordance with the local legislation and institutional requirements. Written informed consent for participation was not required for this study in accordance with the national legislation and the institutional requirements.

## Author contributions

NL and TT co-authored the manuscript draft with critical revision by KH, JH, and HI. Study was conceptualized and designed by NL, TT, and KH. Approval of data collection by KH. Data was acquired by NL and JH. Data was analyzed by NL. All authors contributed to the article and approved the submitted version.

## Funding

This study was supported by a research grant to NL from Copenhagen University Hospital, Rigshospitalet (No. E-24087-01) and support to TT from the Novo Nordic Foundation (No. 65517). Funding sources were not involved in study design, data collection, manuscript writing, or the decision of publication.

## Conflict of interest

The authors declare that the research was conducted in the absence of any commercial or financial relationships that could be construed as a potential conflict of interest.

## Publisher's note

All claims expressed in this article are solely those of the authors and do not necessarily represent those of their affiliated organizations, or those of the publisher, the editors and the reviewers. Any product that may be evaluated in this article, or claim that may be made by its manufacturer, is not guaranteed or endorsed by the publisher.
