# Supplementary material for: Reducing delay to endovascular reperfusion after relocating a thrombolysis unit
Source: Front Neurol. 2022 Sep 23;13:989607. doi: 10.3389/fneur.2022.989607 (PMC9539547; doi:10.3389/fneur.2022.989607)
Supplement: Supplementary file 1 [file Table_1.DOCX]

| Supplemental table 1 – Additional time metrics | | | | |
| --- | --- | --- | --- | --- |
|  | | Period 1 | Period 2 | p-value |
| Onset to door | | 79 (49-191) | 120 (53-378) | 0.021 |
|  | Known onset | 61 (45-84) | 69 (43-121) | 0.309 |
|  | LSW | 208 (143-486) | 522 (225-749) | 0.009 |
| Onset to needle^††^ | | 85 (65-138) | 102 (68-162) | 0.322 |
|  | Known onset | 74 (62-98) | 88 (64-130) | 0.130 |
|  | LSW | 217 (114-227) | 243 (130-568) | 0.241 |
| Onset to groin puncture | | 210 (171-316) | 237 (139-599) | 0.915 |
|  | Known onset | 180 (166-236) | 150 (120-238) | 0.004 |
|  | LSW | 345 (287-607) | 696 (356-852) | 0.016 |
| Ambulance departure to needle^†^ | | 35 (30-51) | 40 (32-51) | 0.417 |
|  | Known onset | 35 (32-48) | 38 (31-50) | 0.660 |
|  | LSW | 38 (28-58) | 48 (40-54) | 0.599 |
| Ambulance departure to groin^†^ | | 143 (128-175) | 105 (83-139) | <0.001 |
|  | Known onset | 141 (124-162) | 100 (80-130) | <0.001 |
|  | LSW | 161 (138-207) | 115 (89-147) | <0.001 |
| Door to needle^††^ | | 17 (13-26) | 22 (16-32) | 0.022 |
|  | Known onset | 16 (13-24) | 19 (15-28) | 0.080 |
|  | LSW | 19 (11-32) | 26 (22-38) | 0.059 |
| Door to groin puncture | | 126 (110-155) | 86 (65-119) | <0.001 |
|  | Known onset | 121 (108-143) | 79 (61-119) | <0.001 |
|  | LSW | 135 (114-198) | 99 (72-119) | <0.001 |
| Door to reperfusion | | 183 (144-240) | 129 (96-174) | <0.001 |
|  | Known onset | 172 (143-242) | 119 (88-164) | <0.001 |
|  | LSW | 199 (164-236) | 140 (115-187) | <0.001 |
| Imaging to needle^††^ | | 8 (5-15) | 9 (5-17) | 0.556 |
|  | Known onset | 8 (4-15) | 8 (5-15) | 0.586 |
|  | LSW | 9 (7-15) | 14 (6-22) | 0.397 |
| Imaging to groin puncture | | 116 (101-148) | 70 (51-93) | <0.001 |
|  | Known onset | 113 (99-135) | 64 (49-89) | <0.001 |
|  | LSW | 125 (104-163) | 79 (56-99) | <0.001 |
| Values are in minutes; median (IQR).  In period 1, 48 patients (65.8%) had known time of onset and 25 patients (34.2%) had onset registered as time of patient last seen well (LSW). For period 2, the proportions were 107 (59.4%) and 73 (40.6%), respectively.  ^†^Ambulance departure missing for seven patients (all in period 2).  ^††^For patients treated with IVT; 49 patients (67.1%) in period 1 and 82 patients (45.6%) in period 2. | | | | |
